# The Impact of Taxation Reduction on Smoking in Youth between 1990 and 1999: Results from a Reconstructed Cohort Analysis of the Canadian Community Health Surveys

**DOI:** 10.1371/journal.pone.0093412

**Published:** 2014-04-03

**Authors:** Nicholas J. Birkett

**Affiliations:** Department of Epidemiology and Community Medicine and the R.S. McLaughlin Centre for Population Health Risk Assessment, University of Ottawa, Ottawa, Ontario, Canada; University of Tolima, Colombia

## Abstract

**Background:**

Increases in taxation can contribute to smoking control. In the early 1990’s, tobacco smuggling rates in Canada increased dramatically. Governments responded with a substantial reduction in taxes on tobacco products. This study examines the impact of these tax changes on smoking in youth in Canada.

**Methods:**

Data on smoking from three consecutive cycles of the Canadian Community Health Surveys were combined and analyzed using a reconstructed cohort approach. Age, sex and calendar year specific rates of smoking experimentation and the onset of daily smoking were estimated for youth. Estimates apply to the entire Canadian population.

**Results:**

There was a strong increase in smoking in youth in the years following the reduction in tobacco taxes. The increase was stronger in women. The rates returned to pre-1990 rates by about 2002. The number of excess daily smokers for people born between 1977 and 1985 that can be linked to the taxation reduction is about 190,000.

**Interpretation:**

There is strong evidence that reduction of tobacco taxes to combat smuggling had an adverse impact on smoking rates in youth.

## Introduction

Increasing tobacco taxes is an effective method to reduce smoking rates [1]–[3]. This was a core component of the smoking reduction strategy adopted during the 1980’s by both the federal and provincial governments in Canada. Between 1980 and 1993, total taxes on cigarettes were raised by about 600% [4]. Smoking prevalence dropped consistently through-out this period in all age and gender groups.

By the late 1980’s, the tobacco industry in Canada had developed a scheme to avoid paying government taxes on many tobacco products. This involved smuggling billions of cigarettes through a native reserve straddling the Canada-USA border [5]–[8]. The smuggled cigarettes were substantially cheaper than legal cigarettes. This had the effect of reducing the actual price of cigarettes to the public. Smuggling emerged as a major issue in 1990/1. In response, the federal government cut its excise tax by about 50% (from $10.36 to $5.36 per carton of 200 cigarettes) in February 1994 [4], [9]. Five provinces dropped their provincial tax rates as well. By April 1994, the combined federal and provincial cuts had reduced tax rates in these provinces by between $14 and $21 per carton, leading to a nearly 50% reduction in price [4].

Health care professional groups and anti-smoking prevention advocates expressed concern that the cuts in taxation would have negative health impacts. In particular, the cuts made cigarettes more affordable for teenagers, the primary age group during which the cigarette smoking habit becomes established [3]. Stephens [10] provided data on tobacco consumption in Canada between 1990 and 1994 which showed a marked increase in the sale of contraband cigarettes between 1991 and 1993 and that the total amount of tobacco consumed increased in 1994. Gabler et al [9] reported that, in late 1994, legal cigarette sales rose by 51% in Ontario and by 175% in Quebec following the tax rollbacks. In 1997, Hamilton et al [11] reported an analysis of the Survey on Smoking in Canada that was conducted every three months between January 1994 and February 1995. Among people aged 15 years or older, the prevalence of smoking decreased across the whole country during this 15 month period. However, they found evidence that provinces in which the tobacco taxes had been cut had a lower rate of decrease of smoking prevalence and higher rates of starting regular smoking by non-smokers. Additional studies have provided support for an adverse effect of the changed in the early 1990’s on tobacco usage [4], [12], [13]. The results have recently been questioned by Ouellet [14] who reported finding no meaningful change in smoking rates during 1994/5. This publication, which was sponsored by the tobacco industry, has been seriously challenged [15], [16]. However, in the light of the on-going controversy, further examination of novel data is needed.

This report will re-examine the impact of price reductions on youth smoking rates in the 1990’s using an interrupted time-series design in a reconstituted cohort [17], [18] created from three cycles of the cross-sectional Canadian Community Health Surveys.

## Methods

The data for this analysis combines the results from three cycles of the Canadian Community Health Surveys (CCHS): CCHS 2.1 (2003), CCHS 3.1 (2005) and CCHS 4.1 (2007–8). The three cycles used similar sampling methodology and an identical series of questions to obtain information about smoking history and key demographic information. Detailed methodology can be obtained from the Statistics Canada web site: http://www.statcan.gc.ca/. Access to these data sets is available through Statistics Canada (see Appendix S1).

Briefly, the CCHS was a cross-sectional survey with a target population of all people living in Canada over age 12, excluding people living on Indian reserves, some remote regions, members of the Canadian Armed Forces and institutional residents. It covered about 98% of the total Canadian population. Stratified area-sampling methods based on the Canadian Labour Force survey were used to identify the candidate respondents. One person normally resident in each selected household was chosen to participate in the survey. Differential probability of selection into the sample is adjusted in analysis through weighting. Interviews were conducted in person or via telephone. About 2% of interviews were conducted with a proxy respondent since the targeted respondent was unavailable for interview.

The results from the three cycles of the CCHS were combined for this paper. Results from individual surveys were examined and revealed similar patterns to the combined data (results not available for display due to restrictions on data release by Statistics Canada). The weighting within each individual survey was maintained for all analyses. The three surveys were combined by treating them as super-strata. It is possible that a few individuals (<0.5%) participated in more than one CCHS cycle. Statistics Canada does not have information on the extent of overlap.

The CCHS has a complex smoking section. It first determines if a person is a current daily smoker, a current occasional smoker or a current non-smoker. Then, different sections of the questionnaire are asked of these three groups. Where appropriate, information was collected about the time when the person first experimented with smoking, first smoked on a daily basis and when they stopped smoking (if they had quit). This information was collected as the reported age. In order to convert age to calendar time, we assigned a date 6 months after their day of birth for the day when smoking was first started. A few subjects who failed to provide birth information were assigned a start date of July 1. The detailed questions used by the CCHS to collect this information can be found in Appendix S2.

A reconstructed cohort approach [17], [18] was used to estimate the age-period specific rates of smoking based on two definitions: initial experimentation and onset of daily smoking. The validity of this approach to estimating historical smoking histories has been established by Bilal et al [19]. For each subject in the survey, a timeline was created starting at birth and ending when they fulfilled the smoking criterion under consideration or the date of survey interview. A lexis-diagram approach was used to accumulate age-period-sex specific person-years at risk and counts of events. Person-time and events were accumulated by examining the estimated status for each subject on each day of their follow-up. For each day of this timeline, the age of the subject was determined based on the calendar year and birth date information. One person-day was accumulated into the appropriate age/calendar year group. If the person met the smoking criterion, then the appropriate event-count group was incremented. The ratio of the number of events and the person-time within each age-period specific group provides an estimate of the rate of smoking experimentation and onset of daily smoking. The estimates were obtained using the ratio estimation tools within Proc Surveymeans in SAS 9.2 (Cary, NC). All analyses were weighted to reflect the sample selection process, using weights provided by Statistics Canada. However, due to the combination of the three surveys and the complex process of creating the reconstructed cohort data, adjustment of the variance estimates for the complex cluster sampling was not feasible. This should lead to some under-estimation of the variances. Caution should be used in interpreting values of marginal statistical significance.

### Statistical Methods

Three analytical approaches were applied to the reconstructed cohort data: Graphical review of incidence rates over calendar time, negative binomial regression and estimation of birth cohort trends.

Age-specific estimates of the rates for the two smoking outcome events were obtained for each calendar year between 1980 and 2003 inclusive. Analyses were limited to events occurring between age 12 and 24. Due to small numbers, age groups 19 and 20 were combined, as were age groups 21 through 24. These estimates were summarized in graphic format.

A count regression modeling approach was used to examine the effects of age and calendar year on the rates of smoking experimentation and onset of daily smoking within the period 1993–1998. Poisson regression models were initially estimated but were rejected due to lack-of-fit from over-dispersion. As a result, negative binomial regression modeling was employed. Several models were examined, with the best fit being obtained with a model containing two factors coded as follows:

1. **Calendar year:** a categorical variable. Each year was coded as a dummy variable, with 1990 as the referent group. Contrast testing was employed to test the null hypothesis that the risks associated with a year between 1993 and 1998 inclusive were the same as the risk in 1990.
2. **Age:** a categorical variable. Each year of age was coded as a dummy variable with age 12 as the referent group;

A birth cohort analysis for subjects born between 1973 and 1990 was used to examine trends by age within cohorts born in the same year. This analysis was restricted to ages 12 through 18 due to sample size limitations. Subjects were excluded if they had not reached age 19 at the time of interview. For each birth cohort, an actuarial life-table was computed to estimate the probability of initial smoking experimentation for each age between 12 and 18 inclusive. A separate analysis examined the probability of becoming a daily smoker.

Birth cohort results were used to estimate the excess number of new smokers created through age 18. The number of people ‘at risk’ in each birth cohort was obtained from Statistics Canada birth data (CANSIM) as the number of births between July 1 and June 30 of consecutive years. The estimates for consecutive years were averaged and rounded to the nearest 100 to estimate the size of each birth cohort. Uncertainty in the estimates of excess number of smokers is created by the lack of a counterfactual base for the time period: we do not know what the smoking rates would have been in the absence of the tax reductions. Since the birth cohort trends were relatively stable for the 1969 through 1976 birth cohorts, an estimate for this counterfactual was obtained by using the mean probability of initial experimentation for these eight birth cohorts. The birth cohorts between 1977 and 1985 were all exposed to the higher rates of experimentation during the age range 12 to 18 for at least a part of their lives. They will be the target to determine the excess numbers.

All regression analyses were performed using SAS 9.2 (Cary, NC). Graphs were produced using Sigmaplot 11.0 (Systat Software).

## Results

The sample size and response rates for the three CCHS cycles are shown in Table 1. The combined sample size was 398,978. The overall response rate for the three surveys was 78.6%. The sample was 54.3% female. Nearly two-thirds of the respondents (253,047 or 63%) reported at least some experimentation with smoking cigarettes. Table 2 shows the distribution (unweighted) of the age when respondents initially began to experiment with smoking; the early and mid-teenage years is the main period for smoking experimentation. It also shows similar results for the age of initial daily smoking. As expected, the onset of daily smoking occurred at an older age than initial experimentation. Overall, about 74% of people who experimented with smoking progressed to daily smoking. These patterns are generally similar for males and females.

**Table 1:** Sample size, response rates and demographic information for CCHS cycles 2.1, 3.1 and 4.1.

	Cycle 2.1	Cycle 3.1	Cycle 4.1	Combined
# households	166,222	168,464	172,709	507,395
# respondents	134,072	132,947	131,959	398,978
Response rate (%)	80.7	78.9	76.4	78.6
Male (%)	61,464 (45.9)	60,910 (45.8)	60,027 (45.5)	182,401 (45.7)
Female (%)	72,608 (54.1)	72,037 (54.2)	71,932 (54.5)	216,577 (54.3)

**Table 2:** Age of Initial Smoking Experimentation and Start of Daily Smoking.

Initial experimentation	Male	Female	All
5–11	12,918 (10.5%)	6,942 (5.6%)	19,860 (8.1%)
12–14	39,008 (31.9%)	35,715 (28.7%)	74,723 (30.3%)
15–19	56,418 (46.1%)	60,320 (48.5%)	116,738 (47.3%)
20–24	10,488 (8.6%)	13,881 (11.2%)	24,369 (10.0%)
25–29	2,254 (1.8%)	3,700 (3.0%)	5,954 (2.4%)
30+	1,238 (1.1%)	3,929 (3.2%)	5,167 (2.0%)
Missing	3,332	2,904	6,236
Total (not missing)	122,324 (100%)	124,487 (100.2%)	246,811 (100.1%)
**Daily smoking**			
5–11	2,633 (2.8%)	1,451 (1.6%)	4,084 (2.2%)
12–14	15,064 (16.2%)	13,864 (15.4%)	28,928 (15.8%)
15–19	55,391 (59.5%)	48,848 (54.4%)	104,239 (57.0%)
20–24	14,375 (15.8%)	16,480 (18.4%)	31,215 (17.0%)
25–29	3,308 (3.6%)	4,468 (5.0%)	7,776 (4.3%)
30+	2,020 (2.2%)	4,672 (5.2%)	6,692 (3.7%)
Missing	2,096	1,942	4,038
Total (not missing)	93,151 (100.1%)	89,783 (100.0%)	182,934 (100.0%)

**Figure 1:**
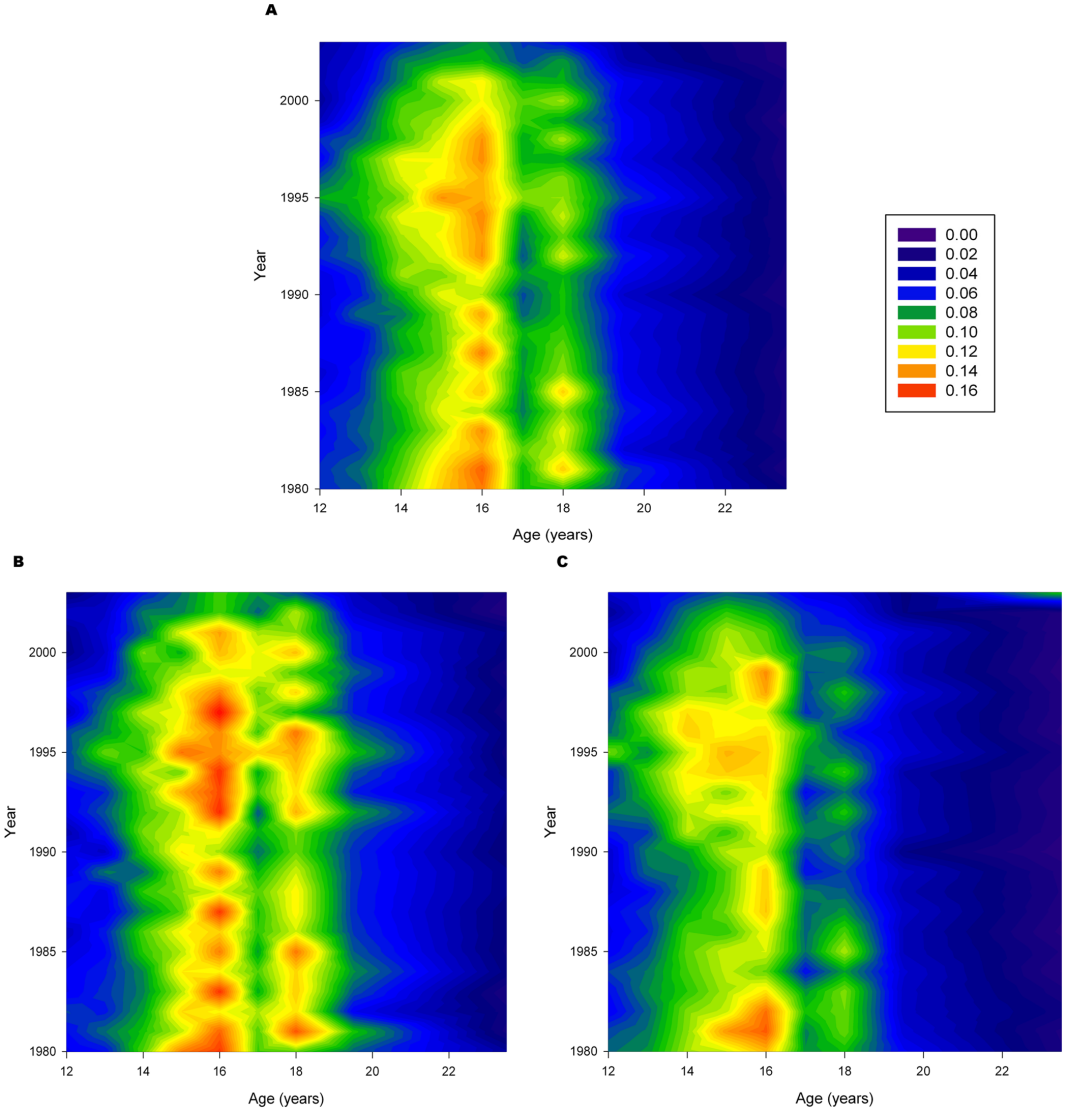
Initial Smoking Experimentation Rates. Rates of smoking experimentation from 1980 to 2003, ages 12–24. Blue colors indicate low rates; red colors indicate high rates. Temporal trends are shown when the color changes along vertical lines through the graphs. (A) Males and females combined. (B) Males only (C) Females only.

Figure 1 presents a two dimensional contour plot to summarize the variation in the rates of initial experimentation with cigarette smoking. The x-axis indexes age while the y-axis indexes calendar year (period). The colors indicate the rate of experimentation: blue indicates low rates while redder colors indicate higher rates. In the absence of rate variation by age, the colors should be the same for horizontal lines across the plot. Similarly, in the absence of temporal trends, the colors should be unchanged for vertical lines.

Initial experimentation was largely confined to the early/mid teenage years with age 15–16 having the highest rates of experimentation. Rates slowly decreased from 1980 to 1990 as shown by the smaller bands of red. However, around 1992, the rates of experimentation in young teenagers increased sharply, peaking in 1995. There was a fairly rapid decrease back to 1990 levels by around 2003. Generally similar patterns are seen when the results are stratified by sex. However, the increase in the 1990’s may be somewhat more pronounced in females.

**Figure 2:**
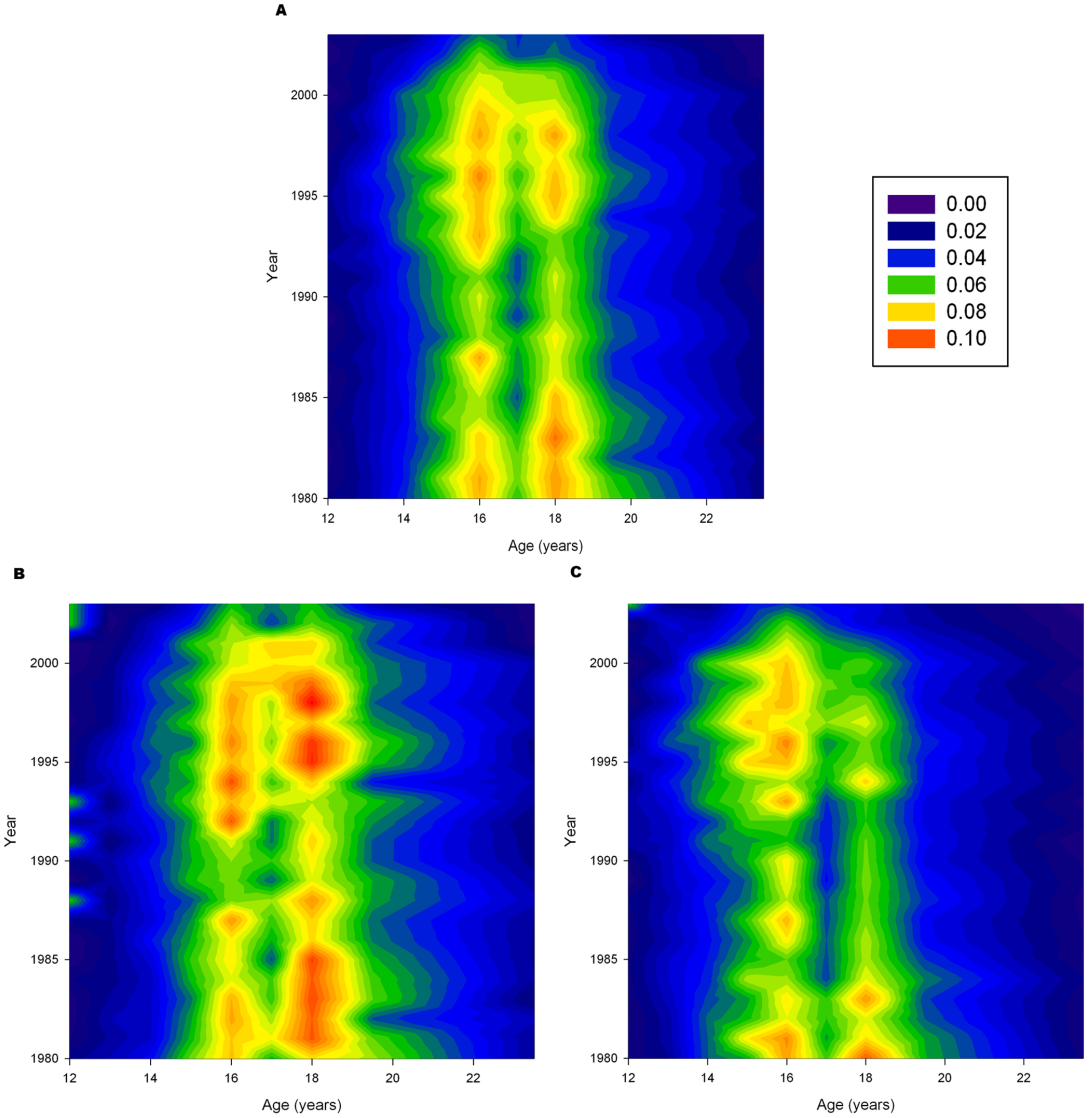
Rates of Onset of Daily Smoking. Rates of onset of daily smoking from 1980 to 2003, ages 12–24. Blue colors indicate low rates; red colors indicate high rates. Temporal trends are shown when the color changes along vertical lines through the graphs. (A) Males and females combined. (B) Males only (C) Females only.

Figure 2 shows similar contour plots for the onset of daily smoking. The onset of daily smoking is again largely a phenomenon of teenagers. There are two peaks for the onset of daily smoking: age 16 and 18. Conversion to daily smoking had been declining in the 1980s as shown by the decrease in the prominence of the red regions between 1985 and 1990. However, starting around 1993, there was a rapid increase in the rate that largely persisted to the end of the study time frame in 2003. Stratifying by sex reveals general similar patterns. The increase in conversion rates for males was more pronounced in 18 year olds. However, for females, the increase was more apparent in 16 year olds.

**Figure 3:**
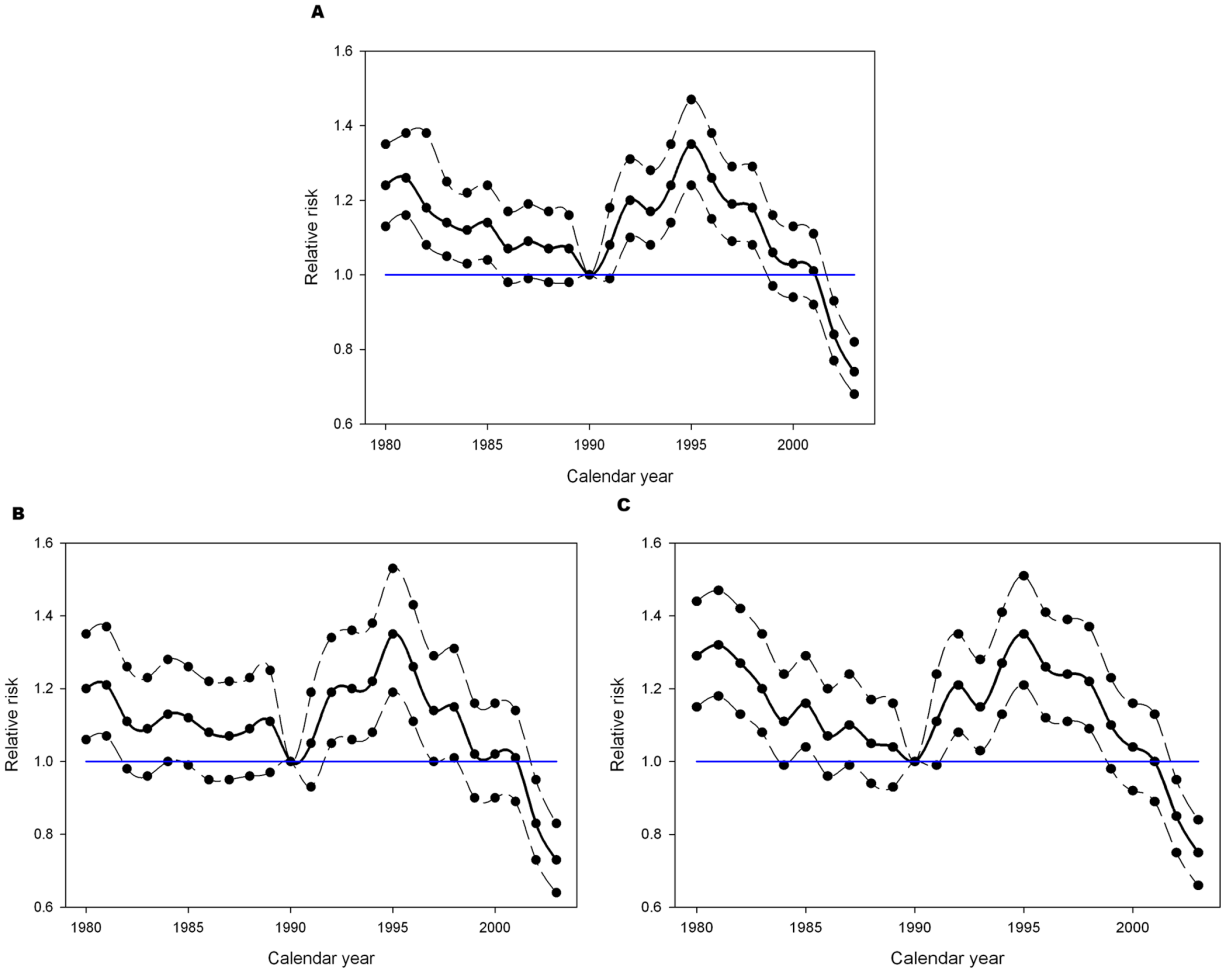
Relative risk of Initial Experimentation with Smoking. Relative risk of initial smoking experimentation between 1980 and 2003. Reference year: 1990. Estimates created using negative binomial regression modeling. Each plot displays the relative risk with approximate 95% confidence intervals. The blue line is the line of null effect (RR = 1.0). The green line shows the estimated smoothed effect between 1993 and 1998. All plots show a gradual decline pre-1990 followed by an increase in the 1990’s which started declining again in the late 1990’s. (A) Males and females combined. (B) Males only (C) Females only.

Negative-binomial regression models were used to estimate the age-specific relative risk of initial smoking experimentation for each year from 1980 to 2003 (Figure 3). The pattern is similar to Figure 1: a decline in risk to about 1990, followed by a rapid up-swing to a peak level in 1995 and a steady decrease up to 2003. The impact of the lowered taxation rates can be seen by examining the estimated smoking experimentation rates for the years 1993 to 1998. Contrast testing found that the rates during these years was significantly higher than the reference year with some evidence that the excess rate varied across the 6 year window (Table 3). The plots in Figure 3 present an estimate for the common effect for the 1993–1998 time period.

**Table 3:** Evidence of Impact of decreased taxation on rates of initial smoking experimentation and onset of daily smoking.

	1993–8 vs. 1990*			1993–8 constant†			1993–8 non-constant¶		
	X^2^	df	p-value	X^2^	df	p-value	X^2^	df	p-value
**Initial Experimentation**									
Male/female combined	43.7	6	<0.0001	29.6	1	<0.0001	14.1	5	0.015
Males alone	23.5	6	0.0006	13.7	1	0.0002	9.8	5	0.081
Females alone	30.6	6	<0.0001	21.4	1	<0.0001	9.2	5	0.10
**Daily Smoking**									
Male/female combined	14.0	6	0.030	9.0	1	0.0028	5.0	5	0.42
Males alone	5.2	6	0.52	1.9	1	0.17	3.3	5	0.65
Females alone	16.2	6	0.013	10.4	1	0.0012	5.8	5	0.33

*Tests the contrast that the estimated rates for the years 1993 through 1998 differs from 1990 (the reference year).

† Tests the contrast that the estimated rate for the years 1993 through 1998 is the same in each year and that this common estimate differs from 1990 (the reference year).

¶ Tests the hypothesis that the annual rates between 1993 and 1998 differ from the estimate common value (the difference between the previous two chi-square values).

**Figure 4:**
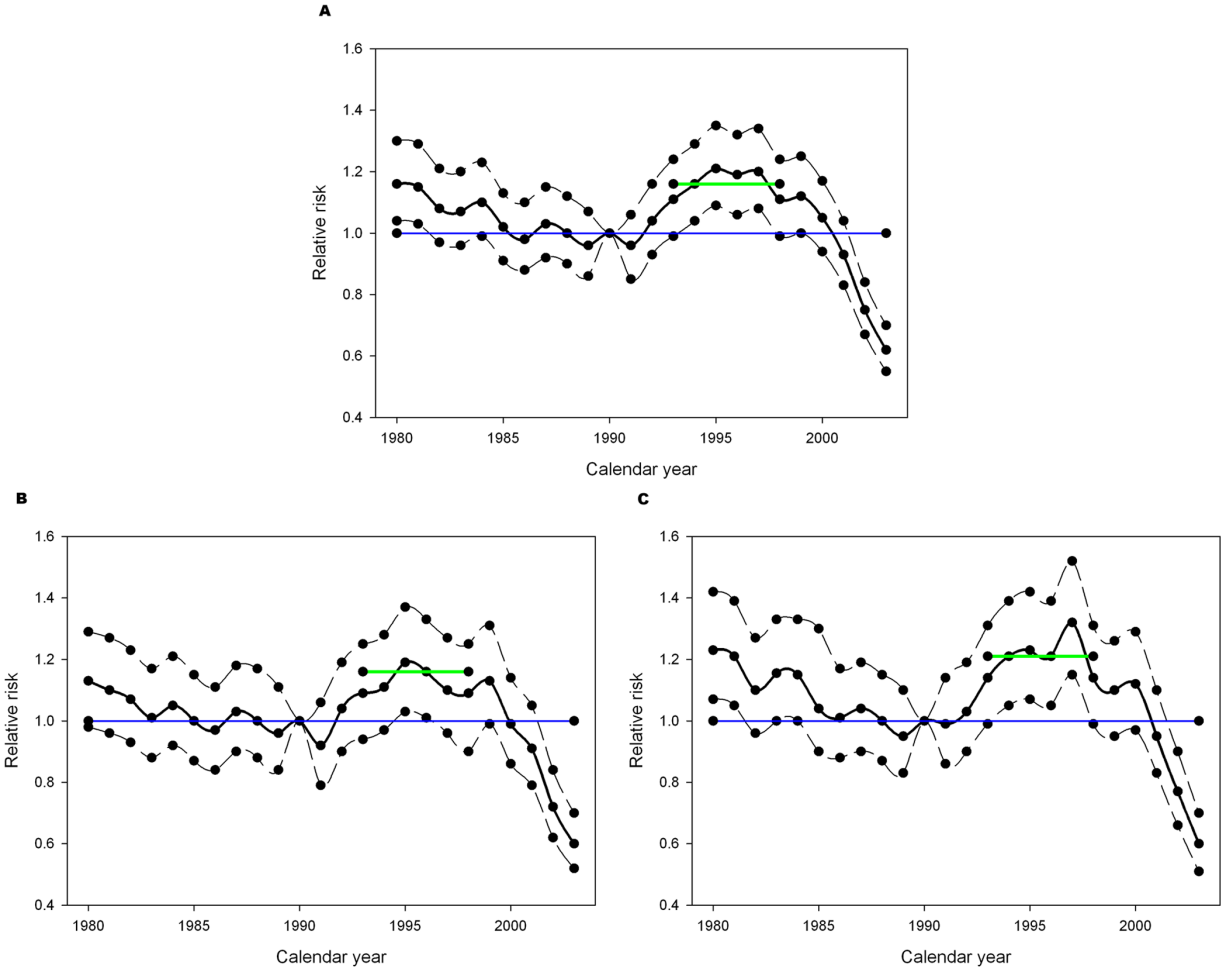
Relative risk of Onset of Daily Smoking. Relative risk of onset of daily smoking between 1980 and 2003. Reference year: 1990. Estimates created using negative binomial regression modeling. Each plot displays the relative risk with approximate 95% confidence intervals. The blue line is the line of null effect (RR = 1.0). The green line shows the estimated smoothed effect between 1993 and 1998. All plots show a gradual decline pre-1990 followed by an increase in the 1990’s which started declining again in the late 1990’s. (A) Males and females combined. (B) Males only (C) Females only.

The relative risk of the onset of daily smoking for each year from 1980 to 2003 is shown in smoothed plots in Figure 4, which also present an estimate for the common effect for the 1993–1998 time period. There is a general decline in risk to about 1990, followed by an upswing to a peak level in 1997 and a steady decrease up to 2003. Compared to smoking experimentation, the peak rate appears delayed by about 2 years. The impact of the lowered taxation can be seen by examining the estimated onset rates for the years 1993 to 1998. Contrast testing found that the rates during these years were significantly higher than the reference year in the male/female combined group and in females alone, but not in males alone. There was no evidence that the excess rate varied across the 6 year window (Table 3).

Table 4 presents estimates of the excess number of teenagers who experimented with smoking for the birth cohorts 1977 through 1985. For males, there was an excess of about 64,400 (95% Confidence interval: 51,000 and 77,700) while for females, the number was higher at about 101,100 (95% CI: 89,400 to 112,700). The excess numbers peaked with the 1979 through 1982 cohorts who would have had the greatest number of years of exposure to the price reductions.

**Table 4:** Excess number of teenagers undertaking initial experimentation, stratified by sex.

Birth Cohort	Size of cohort	Observed probability	‘expected’ probability	Excess number of experimenters (95% CI)
**MALES**				
1977	185,400	0.493	0.457	6,500 (2,400–10,500)
1978	185,600	0.502	0.457	8,400 (4,200–12,500)
1979	187,600	0.525	0.457	12,800 (8,600–16,900)
1980	189,900	0.513	0.457	10,600 (6,200–14,900)
1981	190,900	0.507	0.457	9,500 (5,000–13,900)
1982	191,600	0.517	0.457	11,500 (7,000–15,900)
1983	192,200	0.488	0.457	6,000 (1,600–10,300)
1984	192,900	0.470	0.457	2,400 (–2,200–7,000)
1985	193,300	0.440	0.457	–3,300 (–8,500–1,900)
Total	1,709,400			64,400 (51,000–77,700)
**FEMALES**				
1977	174,700	0.507	0.453	9,500 (6,100–12,800)
1978	175,600	0.507	0.453	9,400 (5,900–12,800)
1979	177,300	0.523	0.453	12,500 (8,900–16,000)
1980	179,800	0.549	0.453	17,200 (13,400–20,900)
1981	181,400	0.519	0.453	11,900 (8,100–15,600)
1982	181,500	0.546	0.453	16,900 (12,900–20,800)
1983	181,900	0.518	0.453	11,800 (7,900–15,600)
1984	182,500	0.505	0.453	9,500 (5,100–13,800)
1985	182,500	0.466	0.453	2,400 (–2,300–7,100)
Total	1,617,200			101,100 (89,400–112,700)

Table 5 presents analogous estimates of the excess number of teenagers who began daily smoking in the birth cohorts 1977 through 1985. For males, there was an excess of about 68,400 (95% Confidence interval: 56,600 and 80,100) while for females, the number was higher at about 124,100 (95% CI: 113,400 to 134,700).

**Table 5:** Excess number of daily smokers to age 18 due to Tax Reductions, stratified by sex.

Birth Cohort	Size of cohort	Observed probability	‘Expected’ probability	Excess number of daily smokers (95% CI)
**Males**				
1977	185,400	0.289	0.248	7,600 (4,000–11,100)
1978	185,600	0.296	0.248	8,800 (5,100–12,400)
1979	187,600	0.298	0.248	9,300 (5,500–13,000)
1980	189,900	0.308	0.248	11,400 (7,500–15,200)
1981	190,900	0.298	0.248	9,600 (5,600–13,500)
1982	191,600	0.303	0.248	10,500 (6,400–14,500)
1983	192,200	0.290	0.248	8,100 (4,200––11,900)
1984	192,900	0.270	0.248	4,200 (100–8,200)
1985	193,300	0.242	0.248	–1,100 (–5,500–3,300)
Total	1,709,400			68,400 (56,600–80,100)
**FEMALES**				
1977	174,700	0.306	0.252	9,300 (6,200–12,300)
1978	175,600	0.299	0.252	8,200 (5,000–11,300)
1979	177,300	0.336	0.252	14,900 (11,600–18,100)
1980	179,800	0.370	0.252	21,200 (17,700–24,600)
1981	181,400	0.329	0.252	13,900 (10,400–17,300)
1982	181,500	0.356	0.252	18,900 (15,200–22,500)
1983	181,900	0.325	0.252	13,300 (9,800–16,700)
1984	182,500	0.327	0.252	13,700 (9,700–17,600)
1985	182,500	0.311	0.252	10,700 (6,300–15,000)
Total	1,617,200			124,100 (113,400–134,700)

## Discussion

The results presented here clearly establish that there was an increase in smoking experimentation and onset of daily smoking among youth during the period between 1992 and 1997. Figures S1 and S2 show that this occurred most dramatically during the mid-teenage years; the effect was noted in both males and females. All of the results were strongly statistically significant. They also reflect a marked departure from the trends in the 1980’s during which time smoking uptake rates either decreased or stayed stable.

The impact of the increase in rates in the 1990’s was striking: about 190,000 people adopted a daily smoking habit who otherwise would not have done so. This effect was more pronounced in females (124,100) than in males (68,400). The analyses do not provide an explanation for the gender difference. One might speculate that the lower costs made smoking affordable for weight control, a known motivator for smoking in female youth [20]. This would be an interesting area for future exploration.

The CCHS studies provide a powerful dataset to explore temporal changes in smoking rates. They are large surveys, which provide sufficient sample size to support year-specific analyses. In addition, unlike smoking specific studies, the smoking questions in the CCHS were contained as part of a much larger interview. The attention of respondents would not have been focused on their smoking answers. This should have resulted in less risk of biased responses being provided.

Reconstructed cohorts rely on the accuracy of the subject’s memory of when key events occurred. Some of these events are inherently imprecise (people don’t start daily smoking on a specific day) or are subject to memory recall issues. It is possible that there could be measurement error in the reported dates and thus that the estimated rates could contain some bias. However, in order to affect the overall conclusions, the recall issues would have had to have been preferentially worse in subjects who were in their teenage years during the 1990’s. There is no reason to suspect that such a pattern occurred. Bilal et al have confirmed the lack of serious bias in a recent comparison of reconstructed smoking rates to rates estimated from contemporaneous data from the same population [19].

Sen and Fatima [4] examined the differential impact of taxation changes in provinces which had the greatest reduction in taxes and confirmed a higher impact in these provinces. For the current analyses, consideration was given to stratifying the current analysis by province. However, the CCHS data set does not contain information about lifetime province of residence. A provincially stratified analysis would have to use current province of residence as a surrogate. Based on the National Household Survey [21], we estimate that about 15% of our subjects would have moved to a new province since the early 1990’s. This could introduce bias into the results. A detailed exploration of the suitability of using surrogate place of residence was beyond the scope of the current study.

Other researchers have explored changes in smoking in Canada during the 1990’s. Hamilton et al [11] reported an analysis of the Survey on Smoking in Canada that provided evidence that the price reduction slowed the decrease in the decline in smoking prevalence demonstrated in the preceding decade. Recently (2010), Ouellet [14] published a re-analysis of the same surveys. In contrast to the Hamilton, he reported little evidence to support any statistically meaningful change in smoking rates during 1994/5 in response to the tobacco tax reductions. This report, which was sponsored by industry with a stake in enhancing tobacco sales, has been subject to serious criticism by Guindon [15] and Pinheiro et al [16]. The analysis method used by Ouellet is non-standard: the outcome states (‘daily smoker’ through ‘non-smoker’) were assigned numerical values (e.g. 1,2,3) from which a mean value was computed for each quarter of the time window between 1994 and 1995 (treating the categorical levels as if they were interval scaled data). Such an analytic approach could mask important changes in the frequency distribution across the three categorical states.

Zhang et al [13] used data from the longitudinal component of the 1994/5 and 1996/7 National Population Health Survey to examine smoking rates within non-smoking youth in 1994/5. They were able to classify subjects by province of residence in 1994/5. The mean price of cigarettes in each province was used to estimate the impact of price reduction on smoking initiation. They found a statistically significantly higher risk of smoking initiation associated with decrease in cost (OR = 1.15 per dollar drop in the cost of 200 cigarettes).

Sen et al [12] examined smoking information from multiple studies conducted in the 1990s (including the Waterloo Smoking Prevention Program, the General Social Survey, the National Population Health Survey, the Youth Smoking Survey and the Canadian Tobacco Use Monitoring Survey). They confirmed the reversal of the trend towards lower rates of smoking in youth that had been observed in the 1980’s.

Gabler and Katz [9] published a comprehensive report for the Fraser Institute which assembled data from multiple sources, including some of the papers cited here, as well as data from Health Canada. Their report includes graphs of taxation levels, cigarette prices and smoking prevalence between 1980 and 2008. These support the analyses presented in this paper. In particular, their figure 17 mirrors the smoking trends reported in this manuscript for youth, including the male/females differences.

The increase in youth smoking in the early 1990’s is clear. The current study does not establish that these changes were necessarily due to the reduction in cigarette prices related to the industry-promoted smuggling operations and subsequent reduction in government taxation rates. However, we have strong circumstantial evidence to support this assertion. The observed increases in smoking rates started at about the time that cigarette smuggling became a prominent issue. The effect was more pronounced during the mid-1990’s after government taxes had been reduced. And, the effect dissipated in the late 1990s’ and early 2000’s when taxation rates were increased to earlier levels [9]. By 1999, excise taxes had been raised by about 30% from the nadir in 1994. Starting in 2001, there was a major increase in taxes, returning close to the 1993 level in inflation-adjusted dollars by 2003 [9]. The taxation increase in 2001 corresponds to the largest drop in experimentation and onset of daily smoking rates that we observed.

Reviewing the history of tobacco control policy in Canada [22] reveals one other policy issue which might have affected smoking rates during this time period. The Quebec ‘*Tobacco Products Control Act*’ was overturned by the Quebec Superior court in 1991 but the court allowed its provision to remain active pending appeal. The Supreme Court of Canada up-held this decision invalidate the Act, but not until 1995. While an adverse impact from this court decision can not be ruled out, any impact would have been expected to occur after 1995, the year when the increase in smoking rates reached its peak. While it is possible that some other societal factor(s) varied along the same time course as the changes in tobacco taxation rates and was responsible for the changes in smoking rates, no such hypotheses have been advanced.

In summary, a reconstructed cohort analysis of three consecutive cycles of the Canadian Community Health Surveys has revealed a striking reversal of smoking rates in the early 1990’s. The rates had been steadily decreasing until about 1992 when they showed a strong increase that reverted to a decrease by the 2000. The trend reversal corresponds to major decreases in the level of tobacco taxation by Canadian governments. This provides evidence to support the adverse effect of reducing tobacco taxes on public health. Maintaining high taxation levels on tobacco products is a significant protective effect on decreasing the rate of smoking uptake among youth.

## Supporting Information

Appendix S1 **Data Availability.**
(DOCX)

Appendix S2 **Original smoking questions used in CCHS.**
(PDF)
