# Supplementary material for: The Impact of Taxation Reduction on Smoking in Youth between 1990 and 1999: Results from a Reconstructed Cohort Analysis of the Canadian Community Health Surveys
Source: PLoS One. 2014 Apr 3;9(4):e93412. doi: 10.1371/journal.pone.0093412 (PMC3974776; doi:10.1371/journal.pone.0093412)
Supplement: Appendix S1 — Data Availability. (DOCX) [file pone.0093412.s001.docx]

## APPENDIX S1: Data Availability

The surveys used in this analysis were designed and executed by Statistics Canada. They provided Public Use Microdata Files for analysis outside Statistics Canada. However, these files only provide a subset of the full data file and do not include the user-specific data required to support the analyses reported in this paper. Access to the full data is allowed by Statistics Canada only under rigorous confidentiality provisions. As such, the author is unable to provide the data for independent analysis. However, interested parties can apply for access through Statistics Canada. The following material, taken from the CCHS User’s Guide [[23](#_ENREF_23)], indicates the options available:

One approach for any user is the production of custom tabulations done by the Client Custom Services staff in Health Statistics Division. This service allows users who do not possess knowledge of tabulation software products to get custom results. The results are screened for confidentiality and reliability concerns before release. There is a charge for this service.

A second approach is the Research Data Centres Program, which allows researchers to submit to Statistics Canada, a research project that uses data from the Master File. These projects are accepted based on a set of specific rules. When the project is accepted, the researcher is designated as a "deemed employee" of Statistics Canada for the duration of the research, and given access to the Master File data from designated Statistics Canada sites. For more information, please consult the Statistics Canada webpage <http://www.statcan.ca/english/rdc/index.htm>.

Finally, the remote access service to the survey master file is another way to have access to these data if for some reason, the user cannot access a RDC. Each purchaser of the microdata product can be supplied with a ‘dummy’ test master file and a corresponding record layout. With this, the user can spend time developing a set of analytical computer programs using the test file to confirm that the program commands are functioning correctly. At that point, the code for the custom tabulations is then sent via e-mail to cchs-escc@statcan.ca. The code will then be transferred into Statistics Canada’s internal secured network and processed using the appropriate master file of CCHS Cycle 3.1 data. Estimates generated will be released to the user, subject to meeting the guidelines for analysis and release outlined in Section 10 of this document. Results are screened for confidentiality and reliability concerns and, once these have been addressed, the output is returned to the client. There is no charge for this service.
