# Supplementary material for: The Impact of Taxation Reduction on Smoking in Youth between 1990 and 1999: Results from a Reconstructed Cohort Analysis of the Canadian Community Health Surveys
Source: PLoS One. 2014 Apr 3;9(4):e93412. doi: 10.1371/journal.pone.0093412 (PMC3974776; doi:10.1371/journal.pone.0093412)
Supplement: Appendix S2 — Original smoking questions used in CCHS. (PDF) [file pone.0093412.s002.pdf]

SMOKING (SMK)

SMK_BEG

SMK_C1 If (do SMK block = 1), go to SMK_QINT.
Otherwise, go to SMK_END.

SMK_QINT **The next questions are about smoking.**
INTERVIEWER: Press <Enter> to continue.

SMK_Q201A
SMKE_01A
In ^YOUR1 lifetime, ^HAVE ^YOU2 smoked a total of 100 or more cigarettes (about 4 packs)?

1 Yes (Go to SMK_Q201C)
2 No
DK, R

SMK_Q201B
SMKE_01B
^HAVE_C ^YOU1 ever smoked a whole cigarette?

1 Yes (Go to SMK_Q201C)
2 No (Go to SMK_Q202)
DK (Go to SMK_Q202)
R

SMK_C201C If SMK_Q201A = R and SMK_Q201B = R, go to SMK_END.
Otherwise, go to SMK_Q202.

SMK_Q201C
SMKE_01C
At what age did ^YOU1 smoke ^YOUR1 first whole cigarette?
INTERVIEWER: Minimum is 5; maximum is [current age].

|_|_|_| Age in years
(MIN: 5) (MAX: current age)
DK, R (Go to SMK_Q202)

SMK_E201C **The entered age at which the respondent first smoked a whole cigarette is invalid. Please return and correct.**

Trigger hard edit if SMK_Q201C < 5 or SMK_Q201C > [current age].

SMK_Q202
SMKE_202
At the present time, ^DOVERB ^YOU2 smoke cigarettes daily, occasionally or not at all?

1 Daily
2 Occasionally (Go to SMK_Q205B)
3 Not at all (Go to SMK_C205D)
DK, R (Go to SMK_END)

Daily smoker (current)

SMK_Q203
SMKE_203

At what age did ^YOU1 begin to smoke cigarettes daily?
INTERVIEWER: Minimum is 5; maximum is [current age].

|_|_|_| Age in years
(MIN: 5) (MAX: current age)
DK, R (Go to SMK_Q204)

SMK_E203

The entered age at which the respondent first began to smoke cigarettes daily is invalid. Please return and correct.

Trigger hard edit if SMK_Q203 < 5 or SMK_Q203 > [current age]

SMK_Q204
SMKE_204

How many cigarettes ^DOVERB ^YOU1 smoke each day now?

|_|_| Cigarettes
(MIN: 1) (MAX: 99; warning after 60)
DK, R

Go to SMK_END

Occasional smoker (current)

SMK_Q205B
SMKE_05B

On the days that ^YOU2 ^DOVERB smoke, how many cigarettes ^DOVERB ^YOU1 usually smoke?

|_|_| Cigarettes
(MIN: 1) (MAX: 99; warning after 60)
DK, R

SMK_Q205C
SMKE_05C

In the past month, on how many days ^HAVE ^YOU1 smoked 1 or more cigarettes?

|_|_| Days
(MIN: 0) (MAX: 30)
DK, R

SMK_C205D

If SMK_Q201A = 2 (has not smoked 100 or more cigarettes lifetime), DK or R, go to SMK_END.

Occasional smoker or non-smoker (current)

SMK_Q205D
SMKE_05D

^HAVE ^YOU1 ever smoked cigarettes daily?

1 Yes (Go to SMK_Q207)
2 No
DK, R (Go to SMK_END)

SMK_C206A

If SMK_Q202 = 2 (current occasional smoker), go to SMK_END.
Otherwise, go to SMK_Q206A.

Non-smoker (current)

SMK_Q206A
SMKE_06A

When did ^YOU1 stop smoking? Was it:
INTERVIEWER: Read categories to respondent.

1	**… less than one year ago?**	
2	**… 1 year to less than 2 years ago?**	(Go to SMK_END)
3	**… 2 years to less than 3 years ago?**	(Go to SMK_END)
4	**… 3 or more years ago?**	(Go to SMK_Q206C)
	DK, R	(Go to SMK_END)

SMK_Q206B
SMKE_06B

In what month did ^YOU1 stop?

1	January	7	July
2	February	8	August
3	March	9	September
4	April	10	October
5	May	11	November
6	June	12	December
	DK, R		

Go to SMK_END

SMK_Q206C
SMKE_06C

How many years ago was it?
INTERVIEWER: Minimum is 3; maximum is [current age] - 5.

|_|_|_| Years
(MIN: 3) (MAX: current age - 5)
DK, R (Go to SMK_END)

SMK_E206C

The number of years ago that the respondent stopped smoking is invalid. Please return and correct.

Trigger hard edit if SMK_Q206C < 3 or (SMK_Q206C > [current age] - 5).

Occasional smoker or non-smoker (current) – Daily smoker (previously)

SMK_Q207
SMKE_207

At what age did ^YOU1 begin to smoke (cigarettes) daily?
INTERVIEWER: Minimum is 5; maximum is [current age].

|_|_|_| Age in years
(MIN: 5) (MAX: current age)
DK, R (Go to SMK_Q208)

SMK_E207

The entered age at which the respondent first began to smoke cigarettes daily is invalid. Please return and correct.

Trigger hard edit if SMK_Q207 < 5 or SMK_Q207 > [current age].

SMK_Q208 SMKE_208 **How many cigarettes did ^YOU1 usually smoke each day?**

|_|_| Cigarettes
(MIN: 1) (MAX: 99; warning after 60)
DK, R

SMK_Q209A SMKE_09A **When did ^YOU1 stop smoking daily? Was it:**
INTERVIEWER: Read categories to respondent.

1	**… less than one year ago?**	
2	**… 1 year to less than 2 years ago?**	(Go to SMK_C210)
3	**… 2 years to less than 3 years ago?**	(Go to SMK_C210)
4	**… 3 or more years ago?**	(Go to SMK_Q209C)
	DK, R	(Go to SMK_END)

SMK_Q209B SMKE_09B **In what month did ^YOU1 stop?**

1	January	7	July
2	February	8	August
3	March	9	September
4	April	10	October
5	May	11	November
6	June	12	December
	DK, R		

Go to SMK_C210

SMK_Q209C SMKE_09C **How many years ago was it**?
INTERVIEWER: Minimum is 3; maximum is [current age] - 5.

|_|_|_| Years
(MIN: 3) (MAX: current age - 5)
DK, R (Go to SMK_C210)

SMK_E209C **The number of years ago that the respondent stopped smoking daily is invalid. Please return and correct.**

Trigger hard edit if SMK_Q209C < 3 or (SMK_Q209C > [current age] – 5).

SMK_C210 If SMK_Q202 = 2 (current occasional smoker), go to SMK_END.
Otherwise, go to SMK_Q210.

Non-smoker (current)

SMK_Q210 SMKE_10 **Was that when ^YOU1 completely quit smoking?**

1	Yes	(Go to SMK_END)
2	No	
	DK, R	(Go to SMK_END)

SMK_Q210A **When did ^YOU1 stop smoking completely? Was it:**
SMKE_10A INTERVIEWER: Read categories to respondent.

1	**… less than one year ago?**	
2	**… 1 year to less than 2 years ago?**	(Go to SMK_END)
3	**… 2 years to less than 3 years ago?**	(Go to SMK_END)
4	**… 3 or more years ago?**	(Go to SMK_Q210C)
	DK, R	(Go to SMK_END)

SMK_Q210B **In what month did ^YOU1 stop?**
SMKE_10B

1	January	7	July
2	February	8	August
3	March	9	September
4	April	10	October
5	May	11	November
6	June	12	December
	DK, R		

Go to SMK_END

SMK_Q210C **How many years ago was it**?
SMKE_10C INTERVIEWER: Minimum is 3; maximum is [current age] - 5.

|_|_|_| Years
(MIN: 3) (MAX: current age - 5)
DK, R (Go to SMK_END)

SMK_E210C **The number of years ago that the respondent completely stopped smoking is invalid. Please return and correct.**

Trigger hard edit if SMK_Q210C < 3 or (SMK_Q210C > [current age] - 5).

SMK_END
